# Supplementary material for: Genomic and Socioeconomic Determinants of Racial Disparities in Breast Cancer Survival: Insights from the All of Us Program
Source: Cancers (Basel). 2024 Sep 27;16(19):3294. doi: 10.3390/cancers16193294 (PMC11475812; doi:10.3390/cancers16193294)
Supplement: Supplementary file 1 [file cancers-16-03294-s001.zip › cancers-3198001-supplementary.pdf]

Supplementary File

Tables

Table S1. List of number of genes available for each chromosome after all steps.

Chromosome Number	Number of Genes	Chromosome Number	Number of Genes	Chromosome Number	Number of Genes
1	866	9	408	17	689
2	694	10	383	18	155
3	573	11	625	19	660
4	318	12	546	20	244
5	449	13	147	21	110
6	459	14	312	22	259
7	419	15	372	X	298
8	324	16	531		

Table S2. Summary of mediation effects on breast cancer survival using Non-Linear Models

Third Variables	Indirect Effect	SD	95% CI	*p*-Value
Age*	−0.074	0.033	(−0.174, −0.047)	<0.0001
General_Health*	0.220	0.049	(0.159, 0.352)	<0.001
General_Quality*	0.041	0.038	(0.001, 0.149)	0.044
Employment_Status	−0.001	0.011	(−0.037, 0.008)	0.409
BMI	−0.031	0.038	(−0.134, 0.020)	0.141
Direct Effect	0.008	0.085	(−0.057, 0.284)	0.510
Total Effect	0.139	0.106	(0.038, 0.459)	0.017

(*) indicate variables with significant indirect effect at 5% level.

Table S3. List of Number of Potential Genomic Middle Variables After Screening in Each Chromosome

Chromosome Number	Number of Genes	Chromosome Number	Number of Genes	Chromosome Number	Number of Genes
1	5	9	8	17	5
2	2	10	2	18	0
3	5	11	8	19	5
4	4	12	8	20	1
5	2	13	3	21	1
6	2	14	1	22	4
7	7	15	1	X	4
8	5	16	10		

Table S4. Indirect Effects of the Significant Genomic Middle Variables from Each Chromosome

Gene Name	Indirect Effect	SD	95% CI	*p*-Value
Model 1: Chromosome 1				
PADI2	0.057	0.039	(0.006, 0.161)	0.026
Model 2: Chromosome 2				
CYP26B1	0.156	0.086	(0.035, 0.0368)	0.013
Model 3: Chromosome 3				
ASB14	0.090	0.046	(0.005, 0.186)	0.036
Model 4: Chromosome 4				
No significant genes found				
Model 5: Chromosome 5				
No significant genes found				
Model 6: Chromosome 6				
RIPK1	0.056	0.030	(0.006, 0.125)	0.038
Model 7: Chromosome 7				
ADCY1	−0.025	0.031	(−0.126, −0.007)	0.021
Model 8: Chromosome 8				
IKBKB	−0.075	0.028	(−0.141, −0.030)	<0.001
Model 9: Chromosome 9				
AL136084.2	0.140	0.068	(0.012, 0.281)	0.031
AL135787.1	0.028	0.019	(0.007, 0.084)	0.004
Model 10: Chromosome 10				
SLC16A12AS1	−0.087	0.042	(−0.196, −0.027)	<0.001
Model 11: Chromosome 11				
ARHGAP32	0.070	0.026	(0.008,0.110)	0.015
Model 12: Chromosome 12				
SLC11A2	0.226	0.082	(0.051, 0.373)	0.009
TMEM132B	0.131	0.054	(0.050, 0.262)	0.002
WBP11	−0.069	0.038	(−0.158, −0.015)	0.015
Model 13: Chromosome 13				
SUPT20H	−0.187	0.104	(−0.460, −0.051)	<0.001
Model 14: Chromosome 14				

No significant genes found				
Model 15: Chromosome 15				
No significant genes found				
Model 16: Chromosome 16				
NARFL	0.062	0.038	(0.012,0.160)	0.021
SALL1	0.064	0.040	(0.018,0.168)	0.007
Model 17: Chromosome 17				
No significant genes found				
Model 18: Chromosome 18				
No significant genes found				
Model 19: Chromosome 19				
No significant genes found				
Model 20: Chromosome 20				
No significant genes found				
Model 21: Chromosome 21				
No significant genes found				
Model 22: Chromosome 22				
AP000345.1	−0.085	0.036	(−0.164, −0.021)	0.005
GNB1L	0.113	0.042	(0.017, 0.180)	0.012
Model 23: Chromosome X				
DCX	0.082	0.029	(0.034, 0.148)	<0.001

Figures

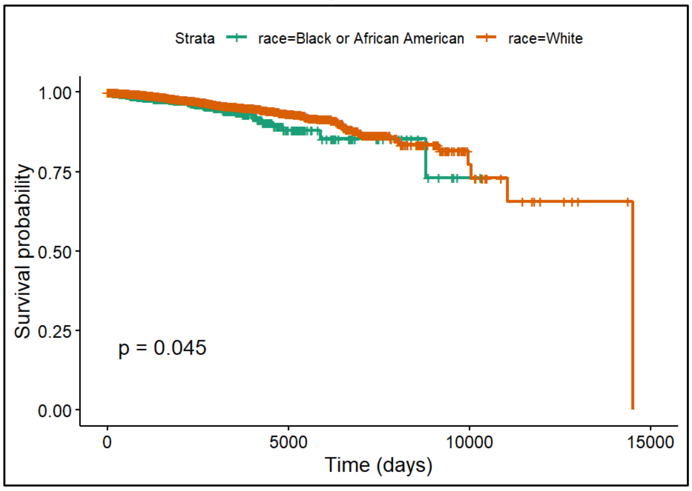

Figure S1. Kaplan-Meier plot for overall survival among breast cancer participants by race.

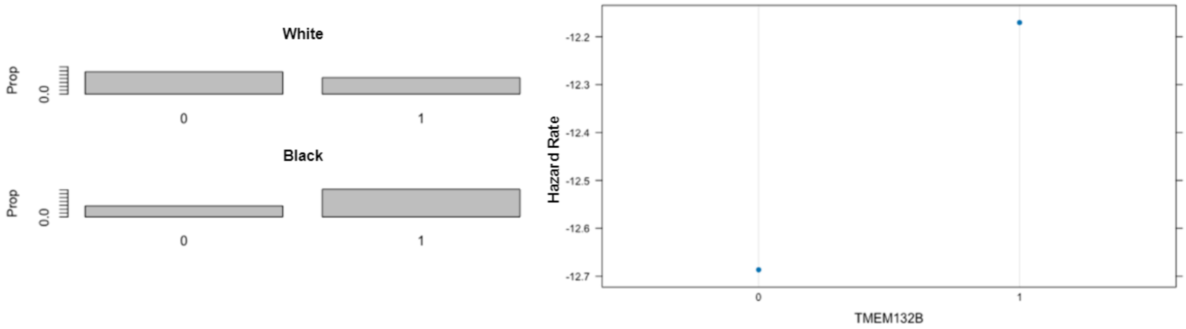

Figure S2. Left: The density of the number of mutations for the gene TMEM132B by race. Right: The breast cancer hazard rate by the number of mutations of gene TMEM132B from Chromosome 16.

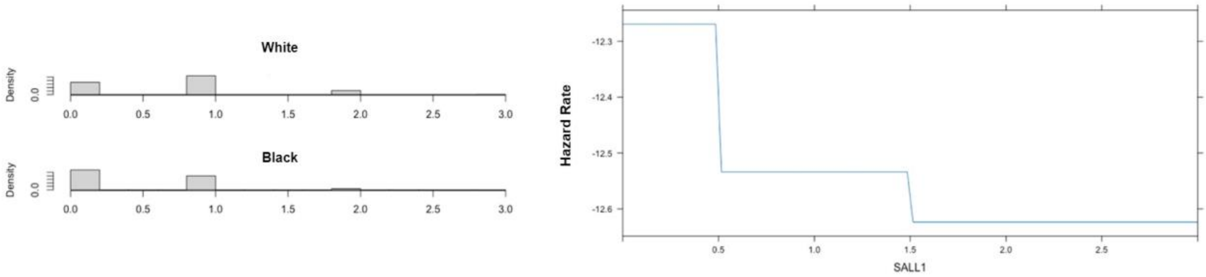

Figure S3. Left: The density of the number of mutations for the gene SALL1 by race. Right: The breast cancer hazard rate by the number of mutations of gene SALL1 from Chromosome 16.

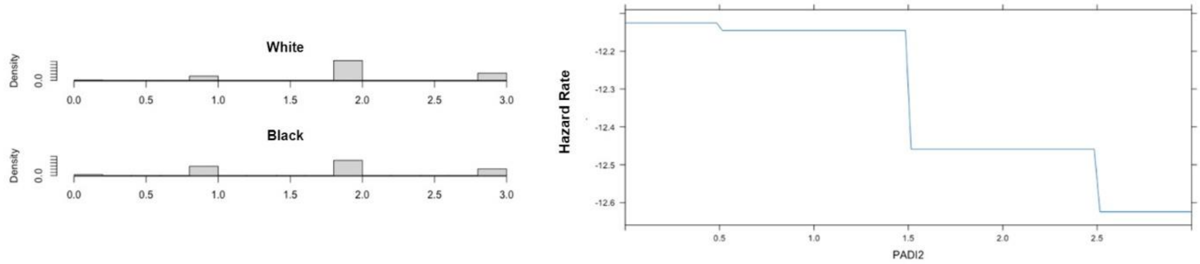

Figure S4. Left: The density of the number of mutations for the gene PADI2 by race. Right: The breast cancer hazard rate by the number of mutations of gene PADI2 from Chromosome 1.

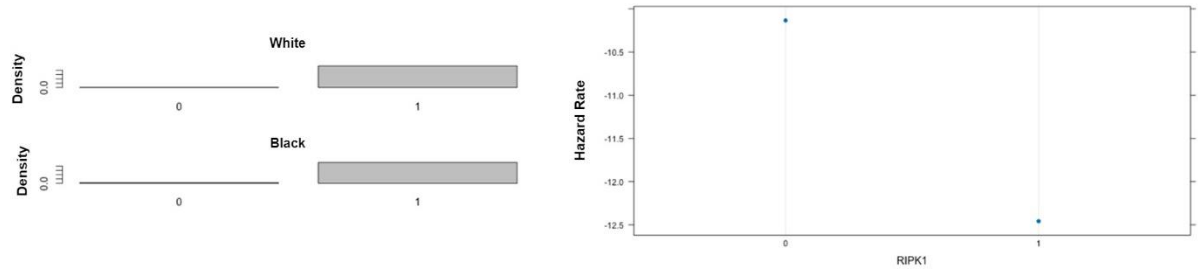

Figure S5. Left: The density of the number of mutations for the gene RIPK1 by race. Right: The breast cancer hazard rate by the number of mutations of gene RIPK1 from Chromosome 6.

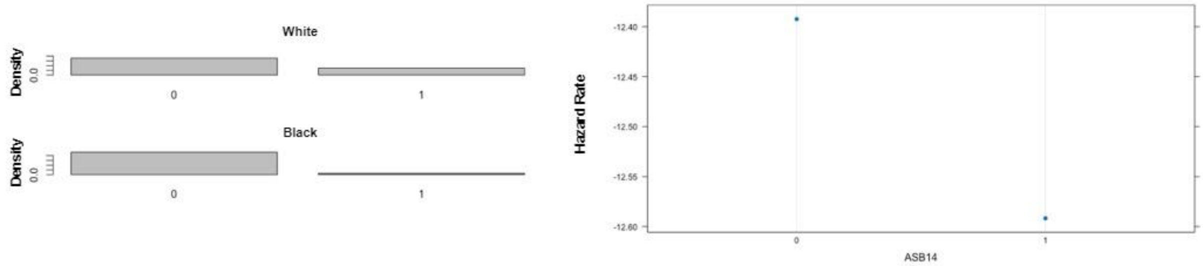

Figure S6. Left: The density of the number of mutations for the gene ASB14 by race. Right: The breast cancer hazard rate by the number of mutations of gene ASB14 from Chromosome 3.

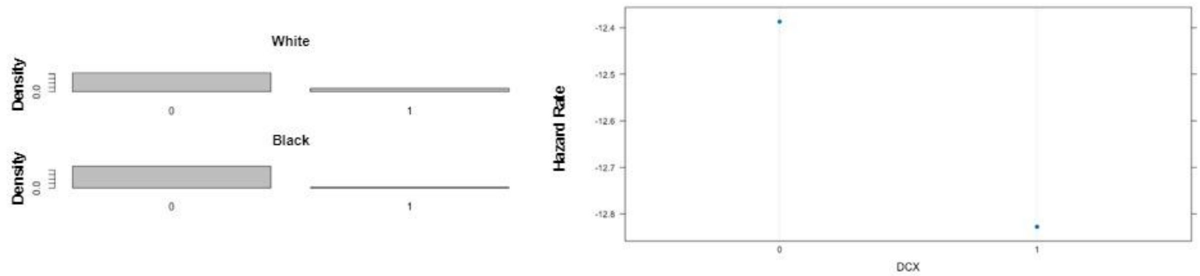

Figure S7. Left: The density of the number of mutations for the gene DCX by race. Right: The breast cancer hazard rate by the number of mutations of gene DCX from Chromosome X.

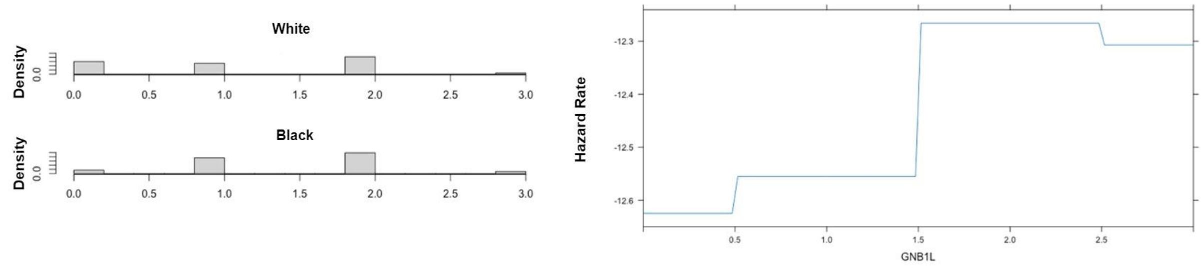

Figure S8. Left: The density of the number of mutations for the gene GNB1L by race. Right: The breast cancer hazard rate by the number of mutations of gene GNB1L from Chromosome 22.

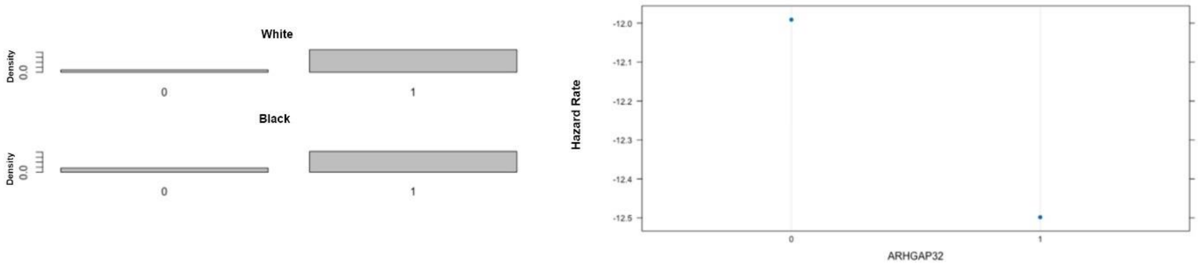

Figure S9. Left: The density of the number of mutations for the gene ARHGAP32 by race. Right: The breast cancer hazard rate by the number of mutations of gene ARHGAP32 from Chromosome 11.

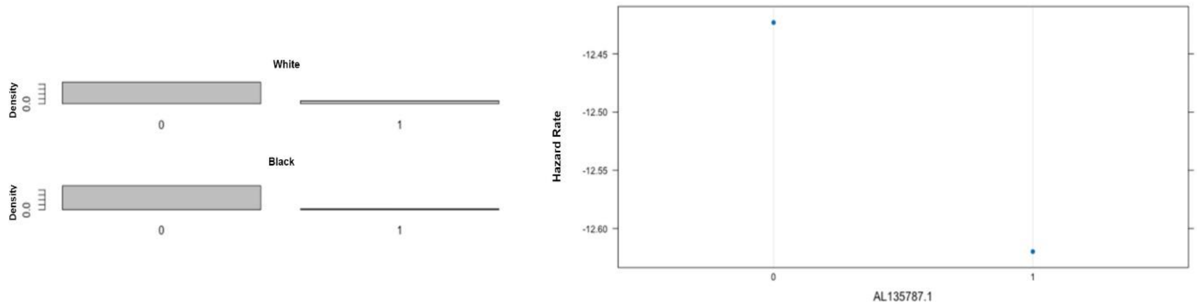

Figure S10. Left: The density of the number of mutations for the gene AL135787.1 by race. Right: The breast cancer hazard rate by the number of mutations of gene AL135787.1 from Chromosome 9.

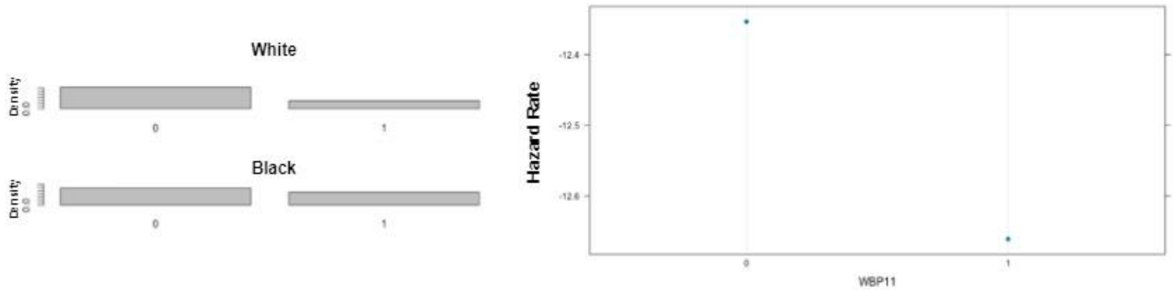

Figure S11. Left: The density of the number of mutations for the gene WBP11 by race. Right: The breast cancer hazard rate by the number of mutations of gene WBP11 from Chromosome 12.

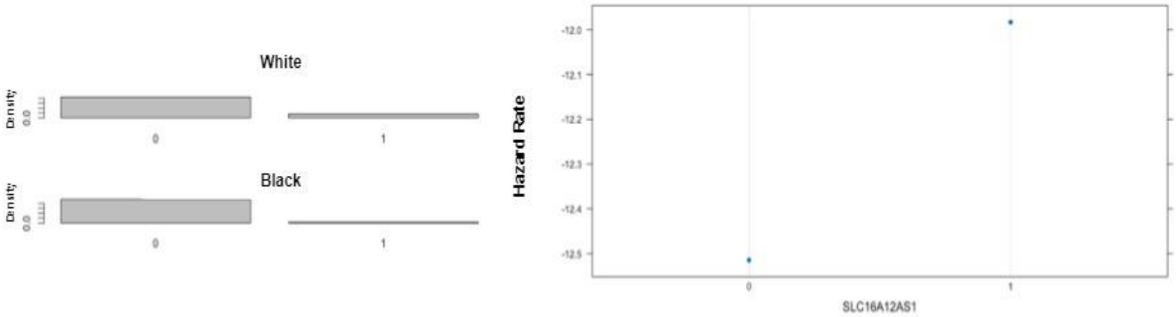

Figure S12. Left: The density of the number of mutations for the gene SLC16A12AS1 by race. Right: The breast cancer hazard rate by the number of mutations of gene SLC16A12AS1 from Chromosome 10.

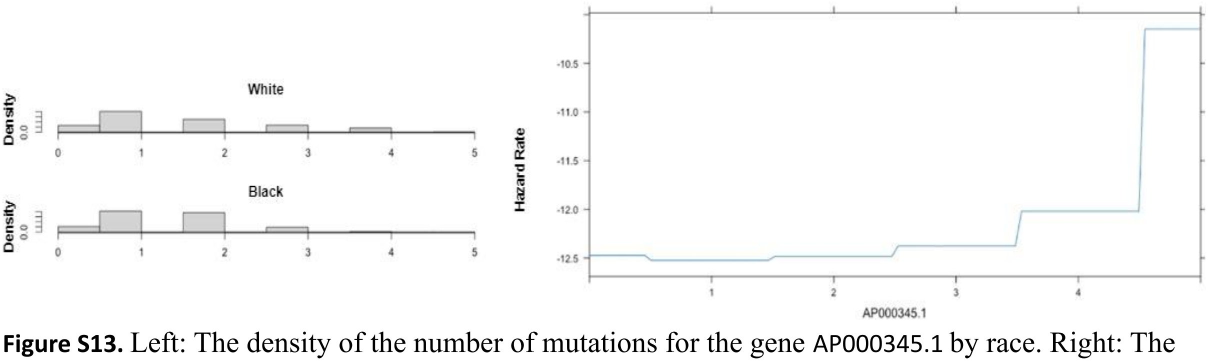

Figure S13. Left: The density of the number of mutations for the gene AP000345.1 by race. Right: The breast cancer hazard rate by the number of mutations of gene AP000345.1 from Chromosome 22.

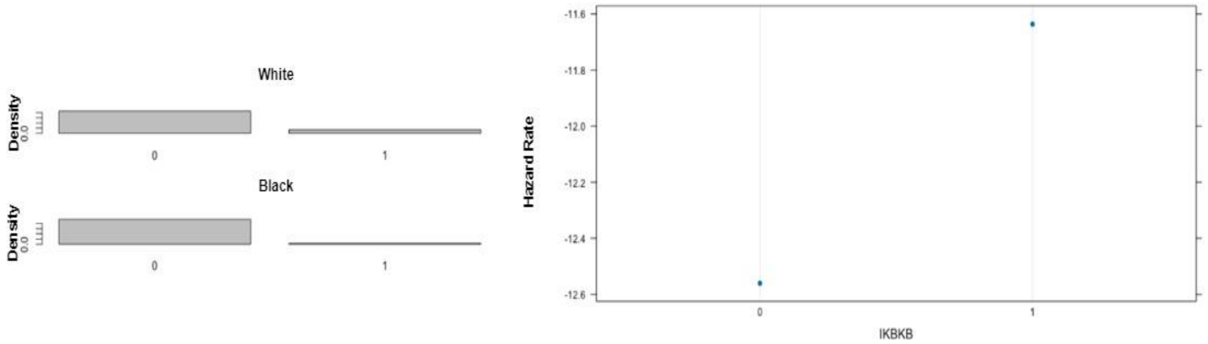

Figure S14. Left: The density of the number of mutations for the gene IKBKB by race. Right: The breast cancer hazard rate by the number of mutations of gene IKBKB from Chromosome 8.

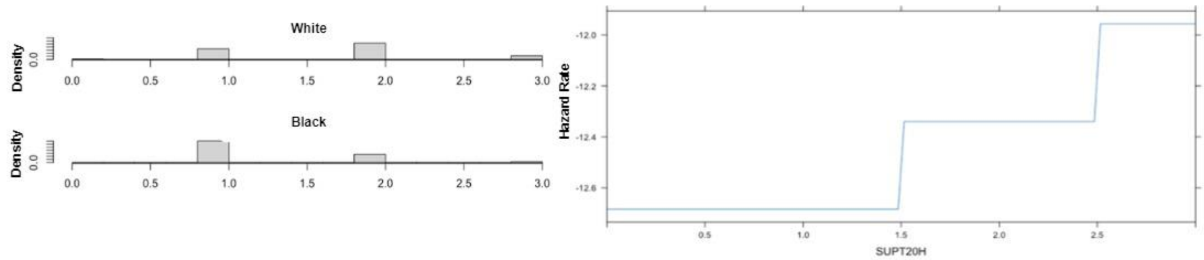

Figure S15. Left: The density of the number of mutations for the gene SUPT20H by race. Right: The breast cancer hazard rate by the number of mutations of gene SUPT20H from Chromosome 13.
